# Usefulness of Stereotactic Body Radiation Therapy for Treatment of Adrenal Gland Metastases

**DOI:** 10.3389/fonc.2019.00732

**Published:** 2019-08-07

**Authors:** Cyrielle Scouarnec, David Pasquier, Joel Luu, Florence le Tinier, Loïc Lebellec, Erwann Rault, Eric Lartigau, Xavier Mirabel

**Affiliations:** ^1^Academic Department of Radiation Oncology, Oscar Lambret Cancer Center, Lille, France; ^2^Department of Biostatistics, Oscar Lambret Cancer Center, Lille, France; ^3^Medical Physics Department - Academic Department of Radiation Oncology, Oscar Lambret Cancer Center, Lille, France

**Keywords:** adrenal gland, metastases, stereotactic body radiation therapy, local control, oligometastatic

## Abstract

**Purpose:** This study aimed to describe our institutional experience in the use of stereotactic body radiation therapy (SBRT) for the management of adrenal gland metastases from multiple primary cancers.

**Materials and Methods:** We retrospectively reviewed 31 patients who underwent SBRT as treatment for 33 adrenal gland lesions in the academic radiotherapy department of Oscar Lambret cancer center between May 2011 and September 2018. The primary study endpoints were 1- and 2-year local control rates, defined as the absence of progression at the treatment site based on the response evaluation criteria in solid tumors (RECIST). Toxicities were graded in accordance with the Common Terminology Criteria for Adverse Events version 4.03.

**Results:** The average tumor volume was 33.5 cm^3^ (standard deviation: 51.7 cm^3^), and the prescribed dose ranged from 30 to 55 Gy given in 3–9 fractions. The median biological effective dose was 112.5 Gy (range: 45–115.5 Gy), assuming α/β = 10. Considering progression at distant sites or death as competing events, the 1- and 2-year actuarial local control rates were 96.5% (95% confidence interval: 84.9–99.7) and 92.6% (95% confidence interval: 79.2–98.7), respectively. According to RECIST, a complete response was achieved in 10 (32.3%) lesions, a partial response in 10 (32.3%) lesions, and stability in 8 (25.8%) lesions. Three patients presented with local relapse at 8.8, 14, and 49.4 months. After a median follow-up of 18 months (range: 4.4–66.4), the median overall survival was 33.5 months (95% confidence interval: 17–not reached), while the median progression-free survival was 7.4 months (95% confidence interval: 3.8–14.1). Treatment-related toxicity was grade 1 or 2 in 42.4% of patients, including nausea (27.3%), abdominal pain (18.2%), vomiting (15.2%), and asthenia (9.1%). None of the patients developed acute grade ≥3 or late toxicity.

**Conclusion:** SBRT seems to be a safe and effective treatment for adrenal gland metastases in patients whose primary tumor and metastatic spread are controlled by systemic treatment. With a 2-year local control rate of 92.6%, SBRT may be considered as one of the first-line treatments in oligometastatic patients with adrenal metastases.

## Introduction

The adrenal glands are a preferential site for metastatic spread, especially from non-small-cell lung cancer, renal cell carcinoma, and melanoma (1).

Improvements in the management of metastatic disease and the widespread use of abdominal computed tomography (CT) and positron emission tomography during staging and follow-up increased the rate of detection of adrenal gland metastases (2, 3).

In this setting, the term “metastatic disease” covers several clinical presentations, ranging from a slowly progressive disease with a limited number of metastases to metastatic efflorescence. In 1995, Hellman and Weichselbaum described the “oligometastatic” concept as a limited metastatic spread in terms of number (maximum of five lesions) (4). In these cases, medical strategies recently evolved into potentially curative treatments, including the multimodal treatments of the primary and metastatic sites.

Adrenalectomy has been the definitive treatment for adrenal gland metastases since several surgical experiences have shown the possibility of long-term survival after resection of isolated adrenal metastasis, especially from non-small cell lung cancer (5–9).

Other local ablative treatments, such as radiofrequency ablation, cryoablation, and transarterial chemoembolization, have reported interesting results in terms of local control in small single institution studies (10, 11).

On the other side, radiation therapy has often been restricted in palliation goals, mostly in cases of abdominal pain (12).

The recent development of stereotactic body radiation therapy (SBRT), which delivers a highly focused ablative dose to the tumor and also spares the healthy surrounding tissues, could probably increase the local control of the oligometastatic disease. This allows the competitiveness of SBRT with other local ablative treatments.

However, only a few data have been published supporting the use of SBRT as treatment for patients with limited metastatic disease involving the adrenal glands. Most of the series concerned few patients, in which the biological effective dose was often <90 Gy (α/β = 10), which is the dose necessary to sterilize most metastases of solid cancers, particularly non-small cell lung cancers (13–15).

Using a biological effective dose of more than 90 Gy, this study aimed to retrospectively describe our single institution experience in the treatment of adrenal gland metastases from various primary cancers in terms of response, local control, overall survival (OS), progression-free survival (PFS), and toxicity.

## Materials and Methods

Eligibility criteria were: patients aged >18 years, with histologically proven primary cancer disease, with metastatic spread evolution controlled by any systemic treatment administered before SBRT planification, with World Health Organization performance status of ≤2, who underwent SBRT delivered as an ablative therapy using a CyberKnife linear robotic accelerator between May 2011 and September 2018, and with no previous surgery or radiation therapy on the adrenal glands. In all patients included in this study, stability or partial response of primary and metastatic sites except the adrenal glands was completed by CT or positron emission tomography (PET) evaluation after previous systemic treatment given according to location and histology of the tumor. We identified the patients from the hospital patient files. All cases potentially eligible were reviewed to confirm the inclusion in the study. All consecutive cases matching with eligibility criteria were finally included in the study.

The study complies with the “reference methodology” adopted by the French Data Protection Authority (CNIL) and patients did not object to the use of their clinical data for the research purpose.

### SBRT Treatment Planning

All treatments were delivered using a high precision CyberKnife linear accelerator, with 6-MV photons.

Treatment simulation was performed on a CT simulator that encompassed a free-breathing four-dimensional CT and a millimeter thickness abdominal CT.

Treatment planning was conducted in the following manner:

1. When possible, patients underwent transdermal implantation of a gold fiducial, which was placed in the center of the metastasis under CT scan control. In these cases, the clinical target volume (CTV) was the same as the gross tumor volume. The planning target volume (PTV) was generated by expanding the symmetric margin to 3–5 mm.
  In these patients, real-time tumor tracking was performed using Synchrony motion management, and respiratory motion of the lesions was compensated during treatment. A correlation model was built between orthogonal x-ray images acquired with regular intervals and the position of light emitting diodes located on the patient's chest obtained by infrared camera. This model was continuously updated and used to move the linear accelerator mounted on the robotic arm, anticipating target location with high accuracy.
2. In other patients, the CTV was expanded to the entire range of tumor motion across the respiratory cycle to create an internal target volume (ITV). This was delineated using a set of 5 CT scans acquired through the course of the respiratory cycle from maximum inhalation to maximum exhalation. The PTV was defined using a uniform 3–5 mm expansion. In those cases, real-time tumor tracking system was not used but respiratory motion management was performed.

The liver, kidneys, stomach, duodenum, spinal cord, and small bowels were contoured as organs at risk.

### Follow-Up and Evaluation of Response to SBRT

Observation time started after the first fraction of SBRT.

The primary study endpoints were 1- and 2-year local control rates, defined as the absence of progression at the treatment site according to the Response Evaluation Criteria in Solid Tumors (RECIST).

The secondary study endpoints were best response to treatment according to RECIST, PFS, OS, and level of toxicity (acute and late) using Common Terminology Criteria for Adverse Events version 4.03.

### Statistics

Descriptive statistics were summarized as average values, median, and range for continuous variables, and as frequencies and percentages for categorial variables.

One- and two-year local control rates were derived from the first day of SBRT with cumulative incidence of local relapse, according to the Kalbfleisch and Prentice method (16), considering death or progression at distant sites without local relapse as competing events.

PFS and OS were estimated using Kaplan-Meier method, from the first day of radiation therapy.

## Results

### Patient Characteristics

Patients' characteristics are summarized in Table 1.

**Table 1:** Patients' characteristics.

**Parameter (*n* = 31)**	**No**.	**%**
**Sex**		
Male	20	64.5
Female	11	35.5
**Age, median (range, yr) 63 (38–80)**		
WHO^*^ performance status		
0	16	51.6
1	14	45.2
2	1	3.2
**Primary tumor site**		
Lung	14	45.2
Melanoma	6	19.4
Kidney	3	9.7
Breast	3	9.7
Hepatocellular carcinoma	1	3.2
Stomach	1	3.2
Bladder	1	3.2
Esophagus	1	3.2
Merkel cell carcinoma	1	3.2
**Total number of metastatic sites (including the adrenal gland)**		
1	8	25.8
2	6	19.4
3	3	9.7
5	1	3.2
>5	13	41.9

WHO^*^, World Health Organization.

All patients matching with eligibility criteria were included in the study, leading to a total of 31 patients and 33 lesions. The median age was 63 years (range: 38–80 years). Two patients with bilateral adrenal metastases underwent SBRT; both lesions were treated at the same time for one patient and at 6-months interval for the second patient.

Sixteen right and 17 left-sided adrenal gland lesions were treated (*n* = 33).

Five adrenal metastases (15.2%) observed in 5 patients were diagnosed <6 months after their primary cancer diagnosis (synchronous metastases). Twenty-eight (84.8%) lesions observed in 26 patients developed after a minimal interval of 6 months (metachronous). In these patients, the median interval from the diagnosis of the primary cancer to the diagnosis of the adrenal metastasis was 16.35 months (range: 5–130.5 months).

Twenty five (75.8%) of 31 patients had metastatic sites other than the adrenal glands. Eighteen (58%) of 31 patients were strictly “oligometastatic” and had up to 5 lesions; about 13 (42%) of 31 patients had more than 5 metastatic sites.

All included patients had their metastatic spread controlled by systemic treatment except for adrenal gland metastasis. Stability or partial response of the primary and metastatic sites except the adrenal glands was confirmed by CT scan in 24 cases, PET in 3 cases, and both in 6 cases.

The average tumor volume was 33.5 cm^3^ (standard deviation: 51.7 cm^3^), and the largest diameter was 38.5 mm (standard deviation: 19.8 mm). The characteristics of cancer and metastatic tumors are summarized in Table 2.

**Table 2:** Cancer and metastases characteristics.

**Parameter (*n* = 31)**	**No**.	**%**
**Laterality of adrenal gland metastasis**		
Right	16	48.5
Left	17	51.5
**Adrenal gland metastasis status**		
Synchronous	5	15.2
Metachronous	28	84.8
**Pain related before SBRT**		
Yes	3	9.1
No	30	90.9
	**Mean (SD)**	**Median (range)**
Tumor volume (cm3)	33.5 (51.7)	13.1 (0.9–278.7)
Tumor largest diameter (mm)	38.5 (19.8)	33 (14–103)
Time from primary diagnosis to adrenal gland metastasis (months) (for metachronous metastases only)	34.5 (32)	16.35 (5–130.5)

*SBRT, stereotactic body radiation therapy; SD, standard deviation*.

None of the patients had a histologic confirmation of their adrenal gland metastasis before SBRT, which were diagnosed after repeated CT scans, which confirmed adrenal gland metastases enlargement according the larger diameter and volume measurements.

### Treatment Planning

Among the 33 lesions, 28 (84.8%) were treated using a tracking system after CT-guided transdermal implantation of gold fiducial. The side effects of fiducial implantation occurred in 3 (10.7%) patients: one had spontaneous hematoma resorption and two had pneumothorax, one of whom required a chest tube insertion.

Five (15.2%) lesions were treated with an ITV.

In 29 (87.9%) of 33 cases, CT planning and treatment were performed in the dorsal decubitus position. In patients treated according to an ITV, an abdominal compression using a belt was used to limit breathing movements.

### Dosimetric Planning

All 31 patients underwent SBRT as ablative treatment, and the spread of all metastatic tumors was controlled by systemic treatment.

Of 33 lesions, 24 (72.7%) were irradiated using a dose of 45 Gy delivered in 3 fractions, on alternate days. In other cases, all SBRT treatment schedules and dosimetric characteristics are presented in Table 3.

**Table 3:** Dosimetric characteristics.

**Parameter**	**Mean**	**Median (range)**
Total dose (Gy)	44.6	45 (30–55)
Fractions (n)	3.7	3 (3–9)
Dose per fraction (Gy)	13.2	15 (5-15)
BED^*^ (Gy)	103.8	112.5 (45–115.5)
Isodose line (%)	82.4	83 (62–90)

BED^*^, biological effective dose.

The median biological effective dose (BED) was 112.5 Gy, assuming α/β = 10, and ranged from 45 Gy for the 5 Gy × 6 fractions schedule to 115.5 Gy for the 11 Gy × 5 fractions schedule.

Among the 33 lesions, 27 (81.8%) were irradiated with BED >100 Gy.

The dose was prescribed to a median isodose of 83% (range: 62–90).

### Clinical Outcome

Treatment outcomes are summarized in Table 4.

**Table 4:** Treatment outcomes and follow-up.

**Parameter**	**No**.	**%**
**Clinical response (RECIST) on adrenal gland metastases (*****n*** **= 31)**		
Complete	10	32.3
Partial	10	32.3
Stable	8	25.8
Progression	3	9.6
**Metastatic relapse (*****n*** **= 31)**		
Yes	23	74.2
No	8	25.8
**Location of metastatic spread (*****n*** **= 23)**		
Nodal	9	39.1
Liver	7	30.4
Lung	6	26.1
Brain	5	21.7
Bone	5	21.7
Contralateral adrenal gland	4	17.4
Other	4	17.4
	**Mean**	**Median (range)**
Time from treatment to local relapse (months)	24.1	14 (8.8–49.4)
Time from treatment to metastatic relapse (months)	10.2	4.5 (0.6–86.2)

*RECIST, Response Evaluation Criteria in Solid Tumors*.

The median follow-up was 18 months (range: 1.4–89.5 months).

**Figure 1:**
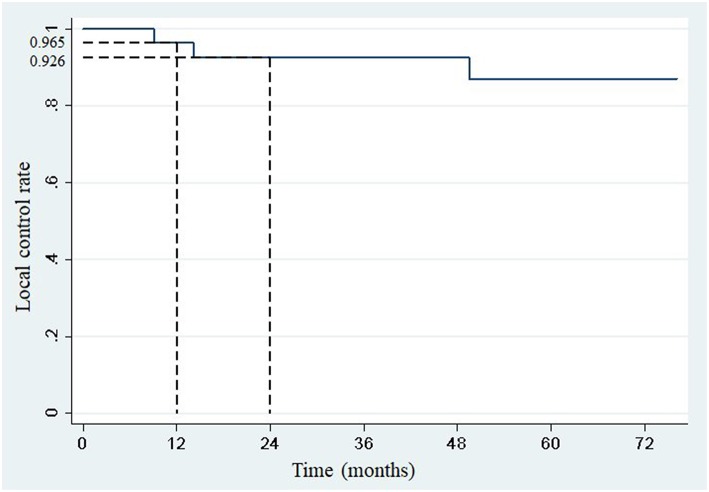
One-year and 2-year local control rates after SBRT.

One- and two-year local control rates were 96.5% (95% confidence interval: 84.9–99.7) and 92.6% (95% confidence interval: 79.2–98.7), respectively (Figure 1). Competing events occurred in 22 patients. The first competing event was progression at distant site in 21 patients, and early death from a pulmonary embolism in one patient. No local relapse was observed after disease progression at distant sites.

**Figure 2:**
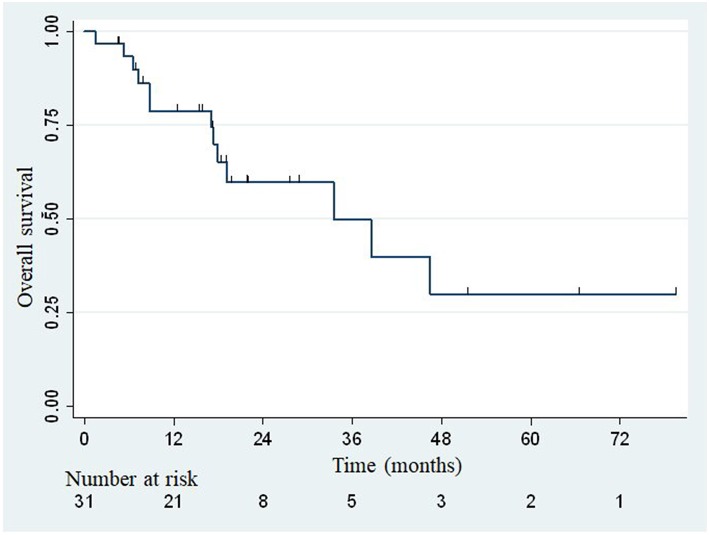
Actuarial Kaplan-Meier overall survival for 31 patients treated with SBRT for adrenal metastases.

**Figure 3:**
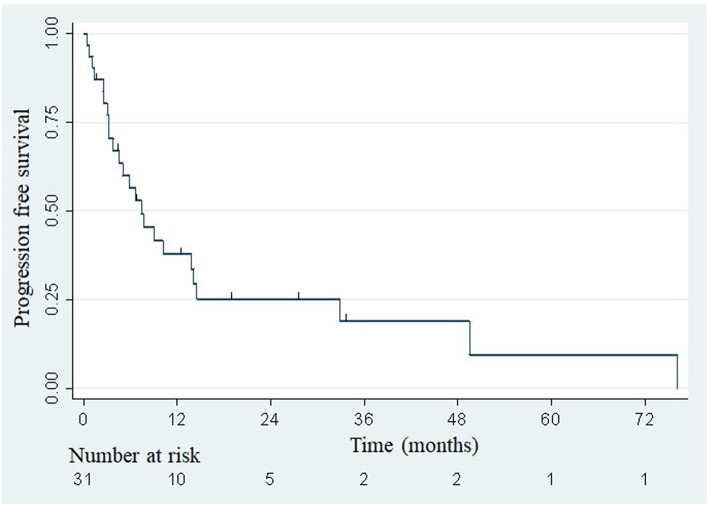
Actuarial Kaplan-Meier progression-free survival for 31 patients treated with SBRT for adrenal metastases.

The median overall survival was 33.5 months (Figure 2), with the lower bound of the confidence interval at 17 months. The upper bound was not defined by lack of events. The median PFS was 7.4 months (95% confidence interval: 3.8–14.1; Figure 3). Of 31 patients, 5 were free from any disease at the last follow-up.

Response to SBRT was evaluated in 31 (94%) of the 33 lesions; one patient died from a pulmonary embolism at day 44, and the other patient had a follow-up period of <3 months.

Abdominal CT was used to evaluate the clinical response of 24 patients (77.4%) and positron emission tomography in 7 patients (22.6%). A complete response was achieved in 10 (32.3%) lesions, a partial response in 10 (32.3%) lesions, and stability in 8 (25.8%) lesions.

At the date of analysis, a local relapse had been reported in three patients (at 8.8, 14, and 49.4 months). These patients were treated with a prescribed dose of 45 gray delivered in 3 fractions (BED = 112.5 Gy) using a real-time tracking system. Primary histologies of those three patients were non-small cell lung cancer in two of them, and hepatocellular carcinoma in the remaining. The median volume of the metastases were 22.3, 50.8, and 56.8 cm^3^. The median volume of PTV receiving the prescribed dose was 87.5% (range: 71.3–98.2). The 3 local failures could be explained by the large volume of the lesions (>50 cm^3^ in 2 cases), and the closeness of the organs at risk in all cases, which led to a degradation of PTV coverage.

In two cases, a partial response could be observed after a new SBRT treatment using the following schedules: 15 Gy × 3 fractions and 6 Gy × 6 fractions. In one case, the patient underwent an adrenalectomy.

At the end of our study, 18 patients were still alive and 13 died of disease progression or intercurrent complication. No deaths related to treatment was reported. The 1- and 2-year OS rates were estimated at 78.8% (95% confidence interval: 58.6–89.9%) and 59.7% (95% confidence interval: 37.2–76.4%), respectively. A distant progression was described in 23 patients, after a median time of 4.5 months (range: 0.6–86.2). The sites of metastatic progression are shown in Table 4.

### Toxicity

Adverse events associated with treatment are shown in Table 5.

**Table 5:** Treatment acute toxicity (*n* = 33).

	**Grade 1**	**Grade 2**	**Total**
	**No. (%)**	**No. (%)**	**(%)**
**Toxicities related in 14 cases (42.4%)**			
Asthenia	1 (3)	2 (6.1)	9.1
Abdominal pain	3 (9.1)	3 (9.1)	18.2
Nausea	5 (15.2)	4 (12.1)	27.3
Vomiting	3 (9.1)	2 (6.1)	15.2
Diarrhea	2 (6.1)	–	6.1

Overall toxicity occurred in 14 (42.4%) of 33 patients. The most common acute side effects were grade 1/2 nausea (*n* = 9, 27.3%), abdominal pain (*n* = 6, 18.2%), vomiting (*n* = 5, 15.2%), and asthenia (*n* = 3, 9.1%). All of them occurred during the days following treatment and improved with symptomatic treatment.

None of the patients developed acute grade ≥3 or late toxicity.

## Discussion

In this study, we reported the outcome of a series of patients treated with SBRT for adrenal gland metastases. Despite a small number of patients and a wide variety of primary tumor histologies, local control could be achieved, with 1- and 2-year local control rates of 96.5 and 92.6%, respectively. No cases of grade 3 or greater toxicity was reported.

With the recent improvements in staging and treatment of most cancers, it is difficult to treat a patient based only on the histologic type or TNM classification. Consequently, the treatment decision relies on the multidisciplinary staff assigned to each patient and is based on the recent status of the primary lesion.

Regarding oligometastatic cancers, the first step to make an appropriate decision is to control the development of metastasis using the most adapted systemic treatment. Therefore, local treatment of the different metastases in various organs should be discussed. In this setting, Gomez et al. reported, in a multi-institutional phase II randomized study, that local consolidative therapy with radiotherapy or surgery improved outcome in 49 patients with oligometastatic non-small cell lung cancer that did not progress after front-line systemic therapy (17). With a median follow up of 38.8 months, the median PFS was 14.2 months in the patients who received local consolidative therapy on all metastatic sites and 4.4 months in the patients assigned to maintenance therapy or observation. A benefit on OS was also found in the local consolidative therapy group (41.2 vs. 17 months).

The results of the multicenter randomized phase II SABR-COMET trial also supported that SBRT delivered on oligometastatic sites from various primary histologies could improve survival (18). Indeed, patients who received SBRT on one to five metastatic sites could achieve a median OS of 41 months, compared to 26 months in patients who received standard of care treatment alone. Additionally, PFS was significantly higher for SBRT-treated patients (12 months) compared to the control group (6 months).

With regard to adrenal metastases, a multicenter French study revealed that the resection of a single adrenal metastasis from a treated lung cancer was associated with a 5-year overall survival of 59% among 46 patients (9).

Although SBRT is not only indicated in patients with a single adrenal metastasis like those reported in surgical case series, data on the results of SBRT are limited (Table 6).

**Table 6:** Comparative summary of the studies reporting on SBRT treatment of adrenal gland metastases.

**Author**	**No. of patients**	**Median dose (range) (Gy)**	**No. of fractions**	**Median BED (range) α/β = 10**	**Local control (%)**	**Overall survival (%)**
Chawla et al. (19)	30	40 (16–50)	4 (4–10)	56 (22.4–75)	1 yr: 55% 2 yrs: 27%	1 yr: 44% 2 yrs: 25%
Holy et al. (20)	18	40 (20–40)	5 (3–12)	65.6 (22.5–72)	1 yr: 94.4% 2 yrs: 78.7%	Median: 21 mos
Torok et al. (21)	7	22 (10–36)	1 (1–3)	51.3	1 yr: 63%	Median: 8 mos
Oshiro et al. (22)	11	45 (30–60)	5 (1,–27)	85.5 (60–132)	6 mos: 94%	1 yr: 55.6% 2 yrs: 33.4%
Rudra et al. (23)	10	36 (24–50)	3 (3–10)	60 (43.2–79.2)	1 yr: 73% 2 yrs: 73%	1 yr: 90%
Casamassima et al. (24)	38	36 (21–54)	3	(60–137)	1 yr: 96% 2 Yrs: 90%	1 yr: 39.7% 2 yrs: 14.5 %
Guiou et al. (25)	9	25 (20–37.5)	5	47	1 yr: 44% 2 yrs: 44%	1 yr: 52% 2 yrs: 13%
Scorsetti et al. (26)	34	32 (20–45)	4 (4–18)	(30–56.3)	1 yr: 66% 2 yrs: 32%	2 yrs: 53%
Ahmed et al. (27)	9	(20–37.5)	5	(28–65.6)	1 yr: 44% 2 yrs: 44%	1 yr: 52% 2 yrs: 13%
Desai et al. (28)	14	54.5 (13–30)	3 (1–5)	42.5 (29–60)	1yr: 64%	1 yr: 87%
Franceze et al. (29)	46	40	4	80	1 yr: 65% 2 yrs: 40%	1 yr: 87%
Haidenberger et al. (30)	23	40.5 (20–45)	3 (1–3)	–	1 yr: 95% 2 yrs: 81%	1 yr: 77% 2 yrs: 72%
Present study (2019)	31	45 (30–55)	3 (3–9)	112.5 (45–115.5)	1 yr: 96.5% 2 yrs: 92.6%	1 yr: 78.8% 2 yrs: 59.7%

Chawla et al. reported in 2008 a 1-year local control rate and OS rates of 55 and 44% in the first series of 30 patients (19). This low local control rate could be explained by the insufficient doses applied, since BED (α/β = 10) ranged from 22.4 Gy (16 Gy in 4 fractions) to 75 Gy (50 Gy in 10 fractions).

In a retrospective study published in 2012, Casamassima et al. reported a series of 38 patients who underwent SBRT on 46 adrenal gland metastases from various origins. A higher BED was administered (range: 60–137 Gy) and was associated with a 1-year local control rate of 96%, weakened by a median follow-up of only 8.1 months and very heterogeneous schedules of SBRT (single-fraction and multi-fraction SBRT) (24).

More recently, Franzese et al. reported in 2016 the outcome of 46 patients, treated with a homogeneous schedule of 40 Gy delivered in 4 fractions (BED 10 = 80 Gy), with a median follow-up of 7.6 months (29). The 1- and 2-year local control rates were 65.5 and 40.7%, respectively. All of these studies confirmed the occurrence of mild toxicity after SBRT.

Considering these results, BED could influence the local control of adrenal gland metastases, regardless of the site of primary cancer.

To the best of our knowledge, this is the only study to use a consistent radiation dose and fractionation, since BED is >100 Gy in 81.8% of cases. This dose escalation is made possible with the use of a real-time tracking of the lesion realized after the implantation of fiducial markers, which minimized the irradiation of adjacent healthy tissues. Respiratory motion management should be recommended whenever possible.

With a median follow-up of 18 months, SBRT seems to be a safe and effective treatment for adrenal gland metastases in patients whose primary tumor and metastatic spread are controlled by systemic treatment.

With 1- and 2-year local control rates of 96.5 and 92.6%, respectively, SBRT may be considered as one of the first-line treatments in oligometastatic patients with adrenal metastases, but also as an alternative to surgery.

## Data Availability

All datasets generated for this study are included in the manuscript and/or the supplementary files.

## Ethics Statement

This study complies with the reference methodology adopted by the French Data Protection Authority (CNIL) and patients did not object to the use of their clinical data for the research purpose.

## Author Contributions

DP, FlT, EL, and XM designed the study and helped supervise the project. CS made substantial contribution to acquisition of data and wrote the paper. ER helped with the collection of dosimetric data. LL and JL performed the analysis and interpretation of the data. All authors discussed the results and commented on the final manuscript.

### Conflict of Interest Statement

The authors declare that the research was conducted in the absence of any commercial or financial relationships that could be construed as a potential conflict of interest.
